# Implementation outcomes of AI documentation support in routine clinical practice: A non-randomized controlled trial

**DOI:** 10.1371/journal.pdig.0001461

**Published:** 2026-07-23

**Authors:** Felix A. Heilmeyer, Lisa Lyssenko, Thomas Reinhard, Christian Haverkamp, Daniel Böhringer

**Affiliations:** 1 Institute for Digitalization in Medicine, University Medical Center Freiburg, Freiburg, Germany; 2 Eye Hospital, University Medical Center Freiburg, Freiburg, Germany; ETH Zurich, SWITZERLAND

## Abstract

While large language models (LLMs) show promise for reducing clinical documentation burden, existing studies rely on simulated environments or require supplemental audio inputs. This study evaluates an AI documentation copilot integrated into real-world electronic health record (EHR) workflows, measuring its impact on physician efficiency and documentation review burden. We conducted a non-randomized controlled trial with 27 ophthalmology clinicians over 12 months (January 2024-February 2025). Participants self-selected into intervention (n = 11) or control (n = 17) groups after a 4-month baseline. The AI system generated inline suggestions for discharge letter conclusions using only the patient’s EHR record as input. The system was based on a Quantized Low-Rank Adaptation (QLoRA) tuned LLM trained on 80,000 institutional records. Primary outcomes included words-per-minute (WPM) rates and the count of required document revisions after checks by the supervising senior physician across 15,615 documents. Intervention group clinicians achieved greater WPM improvements than controls (Δ+7.2 vs. Δ+2.6), while revision frequency decreased within the intervention group (from 1.36 ± 0.14 to 1.26 ± 0.08 per document), with no significant change observed in the control group. Linear mixed-effects modeling confirmed a significant group × exposure interaction for documentation efficiency (p = 0.045), whereas no statistically significant interaction was observed for revision count. This real-world evaluation suggests that AI documentation assistance is associated with improved documentation efficiency under routine clinical conditions, while maintaining documentation standards under established supervision processes. These findings support the cooperative “copilot” design as a viable approach to enhance documentation efficiency without compromising patient safety protocols and indicate its potential to help reduce documentation burden in clinical practice.

## Author summary

Physicians spend a substantial portion of their working hours on electronic health records, often extending documentation tasks into evenings and weekends. This workload contributes to burnout and reduces the time available for direct patient care. Advances in artificial intelligence (AI), particularly large language models, promise to ease this burden, but most evaluations have been restricted to laboratory simulations or required additional data sources such as audio recordings. In this study, we tested an AI documentation assistant directly integrated into the electronic health record system of a university eye clinic. Instead of producing full texts, the system provided short inline suggestions that physicians could accept or edit, keeping them in full control of the final note. Over one year, 27 clinicians participated, with some choosing to use the AI and others continuing their usual workflow. We found that physicians who used AI wrote significantly faster and required fewer corrections by supervisors, without evidence of increased documentation review burden. Our findings suggest that AI assistance is associated with improved documentation efficiency under real-world conditions, which may contribute to reduced documentation burden. By preserving routine oversight and established review processes, this “copilot” approach demonstrates how AI support can be safely and effectively embedded into everyday clinical documentation.

## Introduction

Physicians may spend up to half of their working time interacting with Electronic Health Records (EHR) [1], with a substantial portion of this interaction occurring during after-work hours, weekends, and holidays [2]. This extensive use of EHRs, particularly during non-work hours, has been associated with increased physician burnout [3–5]. Analysis of EHR usage patterns reveals that documentation activities alone account for approximately 25% of the total time physicians spend using these systems [6]. This administrative burden represents a significant challenge to the delivery of healthcare and the well-being of physicians.

Recent advances in Large Language Model (LLM) technology may help alleviate this documentation burden by partially automating clinical documentation with quality matching or even surpassing medical professionals [7–12]. In addition, LLM-generated documentation can improve legibility for patients and family members [11,13,14] and is perceived by some as more empathetic [14]. However, some studies find that the generated text remains susceptible to factual errors and information omissions that could potentially harm patients [15]. Pilot studies with Artificial Intelligence (AI)-assisted documentation suggest the potential to reduce documentation time, freeing up time for patient engagement [11,16–21]. Despite these promising developments, there remains a significant gap in our understanding of how these technologies perform in real-world clinical settings and their impact on physician workflow.

The investigations cited above are conducted with mock data in simulated environments [11,12,18,22], overlooking that clinical documentation is already a highly optimized process with frequent use of templates [23], necessitating evaluation in real-world scenarios. Other studies require audio recordings of physician-patient interactions as additional input data [16,17,19–21], which are not available in many clinical settings.

Our study addresses these limitations by evaluating AI-assisted documentation in an authentic clinical environment rather than simulated scenarios. Unlike previous research, we utilize only routinely recorded EHR data without requiring additional inputs such as audio recordings of patient encounters. This approach ensures broader applicability across diverse healthcare settings where such supplementary data collection may be impractical or unavailable. We specifically designed our system with a collaborative human-AI interface (’copilot’ approach), by providing only inline suggestions instead of full text bodies, which we assume mitigates inaccuracies in AI-generated suggestions. Our primary endpoints measure both the writing speed and the number of document revisions needed as an operational proxy for documentation review burden, providing quantifiable metrics to evaluate the real-world impact of AI-assisted documentation on clinical workflow efficiency and documentation accuracy.

## Methods

### Study design

This non-randomized controlled trial with pre-post-intervention comparison was conducted over a one-year period from January 30, 2024, to February 5, 2025. Following a 4-month baseline period that established pre-intervention documentation patterns, a department-wide trial phase marked the beginning of the intervention period, during which physicians could choose to activate AI assistance at any point and briefly explore the system. This initial trial exposure was not included in the analysis.

The participants were then stratified into two cohorts: The control group comprised clinicians who did not use AI assistance during the observation period, including those who never activated the system and those who did not engage in sustained use beyond the initial trial phase. These clinicians did not use the AI system in a sustained manner during the observation period; accordingly, their analyzed documentation activity reflects routine practice without meaningful AI assistance. The intervention group consisted of participants who chose to activate and continue using AI assistance throughout the study.

For clinicians who did not activate AI assistance during the study period, a reference time point was defined to enable a comparable pre–post analysis. This reference time point corresponded to the midpoint of each clinician’s individual documentation activity (based on cumulative document count) during the observation period.

### Participants

The study was conducted in the outpatient clinic of the Eye Center, Medical Center, University of Freiburg (Germany), which provides specialist care for emergency cases, follow-up evaluations, and routine consultations. Participants comprised 27 junior physicians who were actively involved in clinical documentation in the outpatient setting during the study period. Junior physicians were defined as physicians in postgraduate training who were not yet board-certified and whose clinical documentation was subject to mandatory review and approval by senior physicians.

Eligibility required primary responsibility for generating discharge documentation. All clinical notes were reviewed as part of standard institutional quality assurance procedures, with senior physicians providing feedback and requesting revisions as necessary prior to final approval.

Due to labor law and data protection regulations, individual-level baseline characteristics such as typing speed, workload, or detailed demographic variables could not be collected prospectively as part of the study protocol. To nevertheless explore potential systematic differences between AI users and non-users, we conducted a voluntary retrospective survey among participating clinicians. The survey assessed postgraduate training level, general technology affinity using the validated short version of the German questionnaire “Technikaffinität erfassen” (TA-EG) [24], and attitudes toward AI-supported clinical documentation. Twenty-five of 27 clinicians responded (14 AI users, 11 non-users). Survey results are summarized in S1 Table.

### Intervention

The AI documentation assistant targeted the concluding section of clinical discharge letters, which synthesizes three key elements: (1) primary diagnosis or reason for visit, (2) therapeutic interventions or medications prescribed, and (3) follow-up recommendations. While other parts of the discharge letter contain visit notes and findings recorded during the patient’s visit, clinicians mostly write the concluding section post-consultation, often during after-hours periods.

The system used a custom large language model based on the BLOOM-CLP-German architecture (approximately 7 billion parameters), which was fine-tuned using Quantized Low-Rank Adaptation (QLoRA). Fine-tuning was performed on approximately 80,000 anonymized historical discharge letters from the host institution, covering a broad range of ophthalmologic conditions and visit types.

QLoRA training employed 4-bit NF4 quantization with low-rank adapters applied to the attention layers, enabling parameter-efficient fine-tuning under constrained on-premise compute resources. The detailed model architecture, training procedure, hyperparameter configuration, and evaluation methodology are described in the original publication [22].

**Fig 1:**
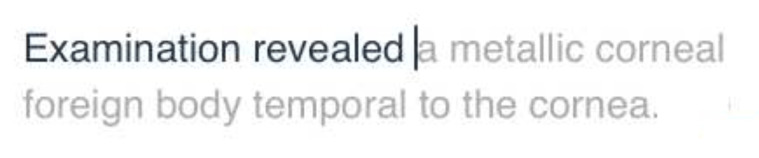
Illustration of the interface of the AI documentation assistant. The implemented “copilot” approach presents AI-generated suggestions informed by the EHR of the patient as inline text (shown in gray) that continues from the physician’s entered text (shown in black). Users can accept suggestions via a configurable keystroke or override them by continuing to type. It is possible to accept the suggestions word by word or as a whole sentence at a time.

Integration occurred directly within the EHR interface through an opt-in checkbox and did not require additional workflow steps. During text entry, AI provided inline suggestions up to a length of one to two sentences through an autocompletion mechanism analogous to consumer systems (e.g., office software assistants). Users accepted proposals via dedicated keystrokes or overrode them through continued typing, maintaining continuous editorial control. Fig 1 illustrates the inline suggestion rendering.

Rather than generating entirely new clinical information, the AI assistant identifies contextually relevant details already documented in the EHR and proposes sentence continuations that meaningfully complete the clinician’s ongoing narrative. An illustrative example is shown in Fig 1, where the AI-generated suggestion integrates examination findings (“Examination revealed … a metallic corneal foreign body temporal to the cornea”) to complete the sentence initiated by the clinician.

Across the intervention period, clinicians accepted, on average, 30.9% of AI-generated suggestions (mean acceptance rate 0.31, SD 0.21), either fully or partially, ranging from 0.02% to 100%, highlighting substantial heterogeneity in AI adoption and use. Suggestions could be accepted word-by-word via a dedicated keystroke or overridden by continued typing. Suggestions were generated incrementally and limited to a single sentence by design, terminating at a sentence boundary marker. Because users could continue typing while suggestion generation was ongoing, the acceptance rate reflects the proportion of generated content incorporated into the final text, rather than guaranteed full visibility of each suggested sentence.

**Fig 2:**
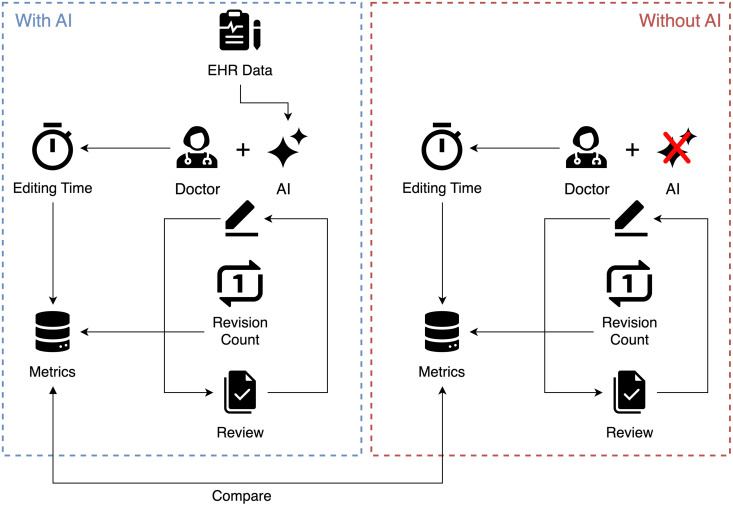
Study design overview comparing documentation workflows with and without AI assistance. The left panel (blue) shows the intervention workflow where physicians received AI-generated suggestions based on EHR data, while the right panel (red) shows the control condition without AI support. Both groups followed identical quality assurance processes, with senior physician review potentially leading to revision requests. Primary outcome measures included documentation efficiency (editing time, converted to words per minute) and documentation review burden (revision count), which were compared between groups.

The design of the intervention specifically avoided the need for additional audio recordings or other external data input beyond standard EHR entries, contrasting with previous ambient documentation approaches. The intervention preserved existing quality assurance protocols in which senior physicians reviewed all the documentation of junior clinicians and sent back documents for revision if necessary. This process was repeated until the documents were approved for publication and archival by the senior physician (see Fig 2).

### Measures

The primary outcomes included documentation efficiency and documentation review burden. Documentation time was measured as active editing duration within the Electronic Health Record (EHR) system’s text editor. Active editing duration was defined as the interval starting from the moment a user activated the editor via a mouse click until the interaction ended.

An interaction was considered finished when the user performed any action not related to text editing, such as switching focus to another element within the EHR, navigating to a different page, or opening an external application. Any time elapsed between the last detected keystroke and editor deactivation was excluded from active editing time.

This approach ensured that idle periods—such as when a clinician finished typing but remained in the editor to speak with a patient or answer a telephone call—were not recorded as active editing time. In addition, clinical workstations automatically lock after 15 minutes of mouse and keyboard inactivity, which also terminates the editor session; these inactivity periods were likewise excluded from editing time calculations.

To account for substantial variance in document length, documentation efficiency was expressed as words per minute (WPM), calculated by dividing total word count by active editor session time. This normalization was chosen to enable comparability across documents of varying length, as document length differed across clinicians and documentation contexts and would otherwise strongly confound comparisons of absolute editing time per document. Documentation review burden was quantified using revision counts, defined as the number of mandatory senior physician review cycles required until document approval (minimum = 1 for approved documents).

### Data aggregation and statistical analysis

Documentation activity varied substantially across clinicians with respect to frequency and timing due to duty schedules, rotations to other clinical units, and differences in full-time and part-time employment. To enable repeated-measures analyses under these conditions, individual documents were aggregated into sequential exposure units (“buckets”), each comprising a fixed number of documents per clinician. This approach preserved the chronological order of documentation activity while ensuring a comparable number of repeated measurements across participants.

Primary analyses were conducted using linear mixed-effects models to account for repeated measurements within clinicians and unbalanced observation patterns. Fixed effects included documentation exposure (“bucket”), group (AI user vs. non-user), and their interaction, with a random intercept for clinicians to account for baseline differences between clinicians.

Separate models were fitted for documentation efficiency and documentation review burden. For documentation efficiency, the dependent variable was documentation efficiency, operationalized as words per minute (WPM) for interpretability, which is inversely related to time per document. For documentation review burden, the dependent variable was revision count, defined as the number of mandatory senior physician review cycles per document.

Although revision count is formally a bounded count variable at the document level, the present analysis was conducted on aggregated exposure units (“buckets”), such that the outcome represented mean revision counts across multiple documents per clinician and time segment. Under these conditions, we used a linear mixed-effects model as a pragmatic and interpretable approach for repeated measures with unbalanced observation patterns.

Formally, the models can be expressed as:


$$\begin{array}{c} \text{Time per document}\sim \text{bucket}\times \text{group}+(1|\text{clinician})\\ \text{Revision count}\sim \text{bucket}\times \text{group}+(1|\text{clinician}) \end{array}$$

Model parameters were estimated using restricted maximum likelihood. Effect sizes are reported as fixed-effect estimates with corresponding 95% confidence intervals. All analyses were performed using Python, including the pingouin statistical package for mixed-effects analyses [25].

### Ethical considerations

This study received ethical approval from the Institutional Review Board of the University of Freiburg Medical Center (registration number: 23–1444-S1). All participants gave formal consent to participate in the study. The clinical documentation underwent mandatory quality review by senior physicians as part of established Standard Operating Procedures (SOPs), ensuring adherence to patient safety protocols and institutional standards. Model training and inference were conducted exclusively on secure on-premise infrastructure, preserving data sovereignty by preventing third-party data transfers. This approach maintained compliance with European Union General Data Protection Regulation (GDPR) requirements for sensitive health information.

## Results

### Participant flow

**Fig 3:**
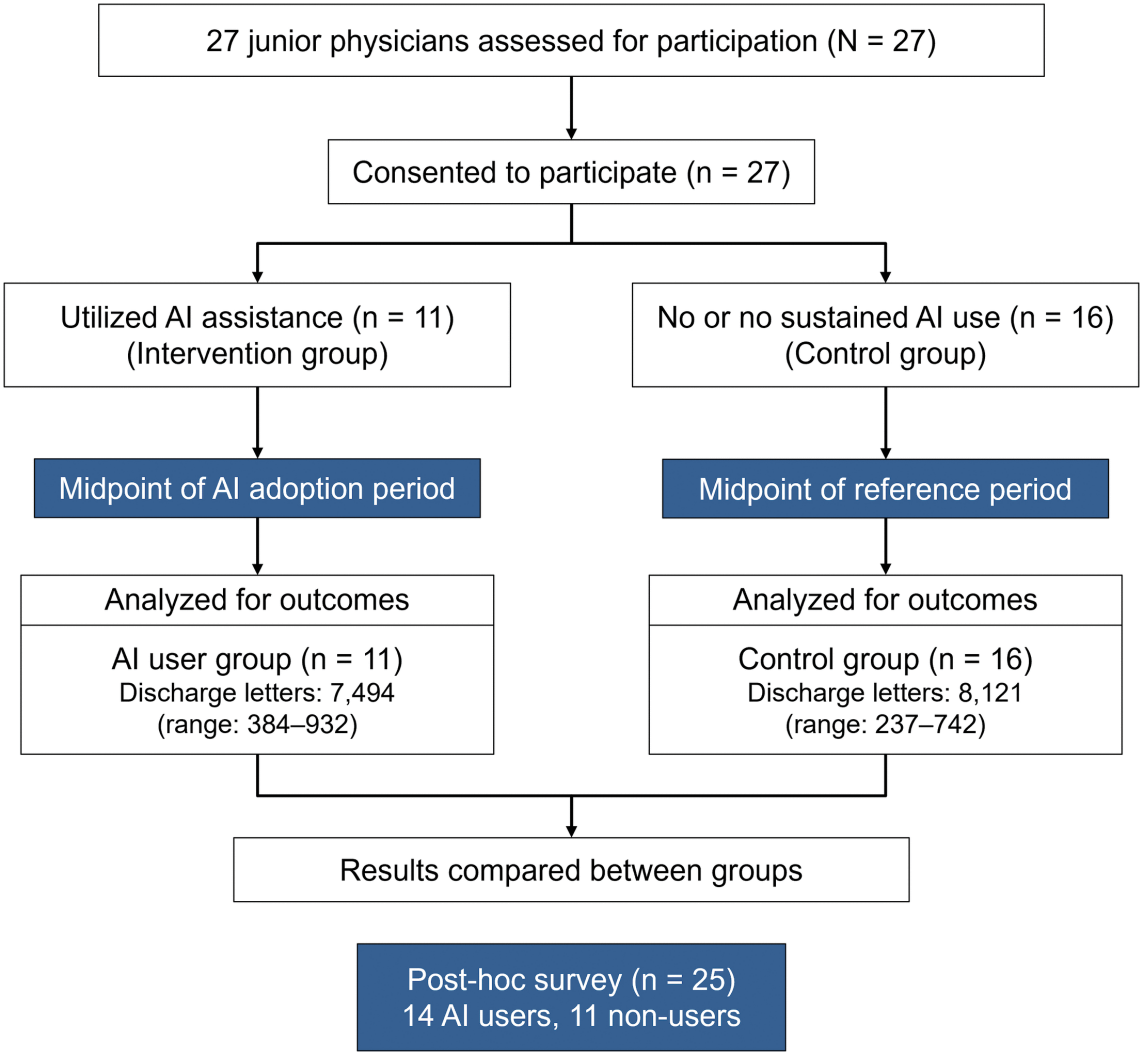
Flow diagram of participation.

Among 27 participating clinicians, 16 were assigned to the control group (3 never activated AI assistance; 13 discontinued use after initial trials), collectively authoring 8,121 discharge letters (range: 237–742 per user). The intervention group comprised 11 clinicians who utilized AI assistance for 44.7–89.5% of their documentation, producing 7,494 discharge letters (range: 384–932 per user) (see Fig 3).

To further characterize participating clinicians, all 27 study participants were invited to complete a voluntary retrospective survey, of whom 25 responded (14 AI users, 11 non-users). Mean postgraduate training level did not differ between AI users and non-users (3.3 ± 1.8 vs. 3.9 ± 1.7 years, p = 0.43). Overall technology affinity, assessed using the TA-EG short scale [24], was comparable between groups (mean score 4.25 ± 0.60 in AI users vs. 3.80 ± 0.74 in non-users, p = 0.12). Among individual TA-EG items, AI users reported a higher willingness to experiment with new technical systems, while no differences were observed for other items. AI users also expressed a more positive attitude toward the usefulness of AI-supported clinical documentation compared with non-users (5.21 ± 1.12 vs. 4.18 ± 1.17, p = 0.036). Detailed survey results are provided in S1 Table.

### Documentation efficiency

**Fig 4:**
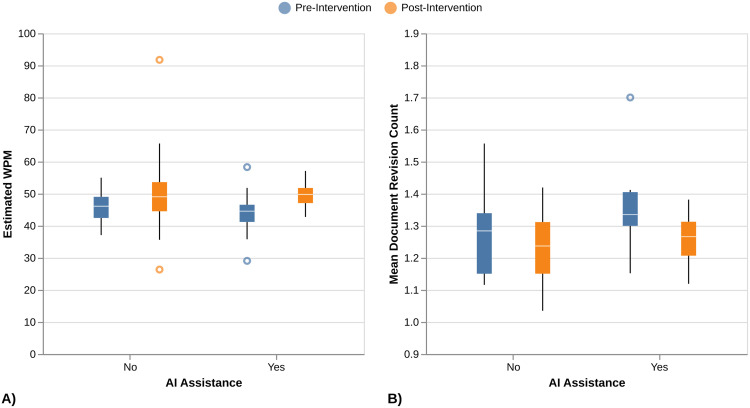
**A)** Documentation efficiency comparison across study groups. Box plots show typing speed (words per minute, WPM) before (blue) and after (orange) the intervention period for control group (left, “No” AI assistance) and intervention group (right, “Yes” AI assistance). The intervention group demonstrated a more substantial WPM increase (Δ+7.2) compared to the control group (Δ+2.6), with a significant group × exposure interaction (*p* = 0.045). Boxes represent interquartile ranges with median lines; whiskers extend to 1.5×IQR; circles indicate outliers. **B)** Documentation review burden comparison across study groups. Box plots show mean document revision counts before (blue) and after (orange) the intervention period for control group (left, “No” AI assistance) and intervention group (right, “Yes” AI assistance). The intervention group demonstrated a significant reduction in required revisions (from 1.36±0.14 to 1.26±0.08, *p* = 0.013), while the control group showed no significant change (1.27±0.16 to 1.23±0.12, *p* = 0.147). Boxes represent interquartile ranges with median lines; whiskers extend to 1.5×IQR; circles indicate outliers.

Pre-intervention documentation efficiency did not differ significantly between groups (two-sided t-test: t = -1.13, p = 0.27), suggesting comparable baseline performance. Descriptively, the control group showed a modest increase from the pre-intervention to the post-intervention period (41.8 ± 20.3 vs. 44.4 ± 20.0 words per minute [WPM]). The intervention group demonstrated a larger increase over the same periods (39.5 ± 20.2 vs. 46.7 ± 19.5 WPM; see Fig 4).

The linear mixed-effects model revealed a significant group × exposure interaction (p = 0.045), indicating that documentation efficiency improved more strongly across documentation exposure units (“buckets”) among clinicians using AI assistance compared with controls. Model-based effect estimates are reported in Table 1.

**Table 1:** Model-based effect estimates.

Outcome	Effect	SS	*F*(1, 25)	p-value	$\eta^{2}$
**WPM**	Group (AI vs. control)	0.014	0.001	0.982	0.000
	Exposure	149.782	31.323	<0.001	0.556
	Group × Exposure interaction	46.664	9.759	0.004	0.281
**Revision count**	Group (AI vs. control)	0.049	2.13	0.157	0.079
	Exposure	0.053	9.52	0.005	0.276
	Group × Exposure interaction	0.010	1.74	0.199	0.065

Abbreviations: SS, sum of squares; df, degrees of freedom; $\eta^{2}$, partial eta squared. Analyses were performed using mixed-design ANOVA models with clinician as the repeated-measures factor.

### Documentation review burden

Descriptively, revision counts decreased within the intervention group from 1.36 ± 0.14 before AI activation to 1.26 ± 0.08 afterward, whereas no meaningful change was observed in the control group (1.27 ± 0.16 vs. 1.23 ± 0.12). In mixed-effects modeling, the group × exposure interaction for revision count did not reach statistical significance, indicating that between-group differences in revision trajectories could not be confirmed.

## Discussion

Physicians using AI achieved an improvement in documentation efficiency nearly triple the gain observed in non-users, accompanied by a within-group reduction in revision requirements among clinicians using AI assistance. These findings suggest that AI writing assistance may help reduce healthcare professionals’ documentation workload under real-world clinical conditions, with potential downstream benefits for clinician well-being and job satisfaction, given the established relationship between documentation burden and burnout [3–5].

Importantly, the observed within-group reduction in revision requirements suggests that AI assistance did not increase supervisory review burden and did not adversely affect documentation under routine clinical oversight, contrary to concerns raised in prior literature regarding the risk of factual inaccuracies in AI-generated medical text [11,15,26]. In particular, both AI users and non-users exhibited natural performance improvements over time, consistent with learning effects; however, the steeper improvement trajectory among AI users may reflect potential additive benefits beyond routine skill acquisition. This aligns with prior work suggesting that AI assistance may support documentation quality and legibility under supervised clinical conditions [7–12].

Beyond overall efficiency gains, our findings suggest that AI assistance may help reduce skill disparities among clinicians. Participants with initially slower documentation speeds were more likely to adopt AI assistance and appeared to derive disproportionate benefits. While we did not systematically examine the influence of factors such as clinical experience, first language, or individual writing styles, these findings highlight the importance of future research into how personal and contextual factors shape AI adoption and benefit distribution.

A key design feature of the intervention was the use of inline AI suggestions with continuous human editorial control. We hypothesize that this interaction model contributed to the maintenance of documentation review burden by mitigating risks associated with fully automated text generation, as previously reported in studies relying on complete AI-generated documents prior to human review [11,15,26]. At the same time, retaining human oversight entails additional cognitive and supervisory effort, and future studies should explicitly examine the trade-off between workload reduction and the cost of sustained human involvement.

Despite the human-in-the-loop design, risks such as automation bias, overreliance on AI-generated suggestions, and potential long-term effects on clinicians’ documentation skills cannot be excluded. In addition, medico-legal responsibility and governance frameworks for AI-assisted documentation remain evolving and were not addressed in this study. These considerations are critical for safe large-scale deployment and warrant systematic investigation in future work.

Finally, successful deployment within a production EHR system extends beyond prior simulation-based studies and demonstrates technical feasibility in routine clinical workflows. While our findings suggest that AI-assisted documentation can be effectively integrated into specific clinical settings, they should not be interpreted as evidence that large language models are ready for unrestricted routine clinical use. Rather, they represent initial real-world evidence obtained under controlled implementation conditions. The focus on a narrowly defined documentation segment further limits conclusions regarding AI support for more complex longitudinal or narrative documentation tasks.

Beyond quantitative efficiency outcomes, and in light of the observed implementation dynamics, the findings of this study underscore the importance of human-centered AI design in clinical documentation support. Consistent with the concept of “augmented intelligence” described by Topol [27], the AI system was implemented as a supportive tool embedded within existing workflows, with clinicians retaining full editorial control and responsibility for clinical content. Close collaboration between clinicians and data scientists during development and deployment enabled alignment with real-world documentation practices and clinical priorities, which likely contributed to acceptance and sustained use. Rather than aiming for automation of clinical decision-making, this approach illustrates how AI can meaningfully reduce documentation burden while preserving professional autonomy and accountability. These principles may be critical for the safe and effective translation of AI systems into routine clinical care.

## Limitations

This study has several important limitations. First, the non-randomized, self-selection design introduces potential confounding, as clinicians who chose to adopt AI assistance may differ systematically from non-users in motivation, documentation practices, or case mix. Although a voluntary retrospective survey did not reveal differences in postgraduate training level or overall technology affinity between groups, AI users reported a higher willingness to experiment with new technologies and a more positive attitude toward AI-assisted documentation. More detailed participant-level characteristics, such as digital literacy or prior experience with AI tools, were not systematically collected due to data protection constraints and the need to maintain feasibility of the voluntary survey. These unmeasured factors may have influenced both AI adoption and observed performance differences.

In addition, baseline differences in documentation efficiency may have influenced the observed effects, as the intervention group showed lower initial WPM, potentially reflecting regression to the mean or a greater potential for improvement. Visit complexity may further influence documentation effort and revision behavior; however, standardized measures of case complexity were not consistently available in the routine EHR data, precluding explicit adjustment for this factor. Residual confounding therefore cannot be excluded, and causal interpretations of the observed effects should be made with caution.

Second, to enable a pre–post comparison among clinicians who did not activate AI assistance, a reference time point based on the midpoint of individual documentation activity was used. This approach was chosen to account for learning effects related to cumulative documentation experience rather than calendar time. However, this design choice introduces potential temporal confounding, as it does not control for seasonal workload variation or institutional factors. Relatedly, the use of aggregated exposure units (“buckets”) represents an additional methodological consideration. Because both the reference time point and the bucket structure are defined based on cumulative documentation activity rather than calendar time, clinicians with different documentation frequencies contribute observations over non-equivalent time intervals. While this approach captures experience-based learning effects, it may limit temporal comparability across individuals. Differences in documentation intensity, duty schedules, clinical rotations, and employment status may therefore introduce residual bias in exposure-related estimates. Alternative time-based sensitivity analyses were not feasible due to substantial inter-individual variability in documentation frequency and timing driven by duty schedules, clinical rotations, and differences in employment status. Furthermore, the study population was limited to junior physicians, and effects may differ for more experienced clinicians with established documentation routines.

Third, documentation efficiency was operationalized as words per minute (WPM), a normalized measure that accounts for variation in document length and enables comparability across heterogeneous clinical cases. In routine clinical practice, absolute editing time per document is strongly influenced not only by document length and case complexity, but also by specialty-specific documentation practices and the clinical setting (e.g., inpatient vs. outpatient care), which vary substantially and would confound comparisons across clinicians and over time. As a result, WPM reflects efficiency of text production relative to document length rather than absolute time expenditure. Accordingly, the findings should not be interpreted as direct evidence of reduced time spent per document. Future studies should complement such normalized efficiency metrics with more granular analyses of absolute time expenditure, ideally in settings where case complexity and documentation requirements can be more explicitly controlled.

Fourth, documentation review burden was approximated using revision counts based on mandatory senior physician review cycles. While this metric reflects supervisory acceptance under routine clinical conditions, it does not directly assess factual correctness, semantic quality, or clinical appropriateness and may be influenced by individual reviewer preferences. Although factual correctness of AI-generated documentation was evaluated in a prior independent validation study using blinded expert review [22], the absence of direct expert-based quality assessment within the present study remains an important limitation. In addition, a Hawthorne effect cannot be excluded, as participants may have modified their documentation behavior due to awareness of being observed.

Finally, the study was conducted within a single specialty (ophthalmology) and focused on a specific section of discharge documentation. Ophthalmology discharge letters often synthesize relatively discrete clinical information, and it remains uncertain whether the observed efficiency gains and maintained documentation review burden would generalize to specialties requiring more complex narrative synthesis or integration of multi-system data. Clinical domains with less standardized narrative structures may require different prompt strategies or exhibit different efficiency gains, underscoring the importance of context-specific evaluation when deploying AI-assisted documentation tools. Future studies should therefore include diverse clinical disciplines to assess broader applicability and required adaptations.

Despite these limitations, our study provides valuable real-world evidence of the benefits of AI assistance - without requiring changes to clinical workflows or additional data collection such as audio recordings - in a production clinical environment, representing an important advance over previous simulation-based studies. These findings justify expanded investigations across medical disciplines, particularly through randomized designs that mitigate the self-selection bias and control for experience-related as well as other confounders.

## Conclusions

This study shows that AI-assisted documentation was associated with more efficient clinical writing while maintaining documentation standards under routine senior physician supervision, as reflected by stable or reduced revision requirements. Together, these findings indicate that appropriately implemented AI assistance may help reduce documentation workload that contributes to physician burnout, without compromising routine quality assurance processes.

At the same time, these results should not be interpreted as evidence that large language models are mature for unrestricted routine clinical use. Rather, they represent initial real-world evidence obtained under controlled implementation conditions.

The “copilot” approach with inline suggestions preserved continuous human oversight while still delivering meaningful efficiency gains, demonstrating that AI assistance can be integrated into existing EHR workflows without requiring additional data collection processes or changes to clinical routines.

Future research should prioritize multi-center validation across diverse clinical environments, inclusion of nursing and other healthcare professionals to assess broader applicability, investigation of personal and contextual factors influencing AI adoption and benefit distribution, and evaluation of long-term effects on clinician satisfaction and practice. Taken together, our findings suggest that AI-assisted documentation technology may be sufficiently mature for cautious, supervised implementation in selected clinical settings, with the potential to return valuable time to clinicians and support care delivery without compromising routine quality assurance.

## Supporting information

S1 Table Raw results of the retrospective survey among participating clinicians. (XLSX)

S1 Data De-identified data on documentation efficiency (words per minute, absolute editing time per document section). (CSV)

S2 Data Documentation review burden: aggregated document revision counts after review by senior physician per participant. (CSV)
